# Network pharmacology-based strategy for predicting therapy targets of Ecliptae Herba on breast cancer

**DOI:** 10.1097/MD.0000000000035384

**Published:** 2023-10-13

**Authors:** Hui Li, Wei Shi, Tingming Shen, Siwen Hui, Manting Hou, Ziying Wei, Shuanglin Qin, Zhaofang Bai, Junling Cao

**Affiliations:** a School of Chinese Materia Medica, Beijing University of Chinese Medicine, Beijing, China; b Department of Hepatology, The Fifth Medical Center of Chinese PLA General Hospital, Beijing, China; c China Military Institute of Chinese Materia, The Fifth Medical Center of Chinese PLA General Hospital, Beijing, China; d Ningde Hospital of Traditional Chinese Medicine, Ningde, China; e School of Pharmacy, Xianning Medical College, Hubei University of Science and Technology, Xianning, China; f Luoyang Branch of Dongzhimen Hospital Afiliated to Beijing University of Chinese Medicine, Beijing, China.

**Keywords:** breast cancer, Ecliptae Herba, molecular docking, network pharmacology, TGF-β1

## Abstract

Breast cancer is a prevalent malignancy affecting women globally, characterized by significant morbidity and mortality rates. Ecliptae Herba is a traditional herbal medicine commonly used in clinical practice, has recently been found to possess antitumor properties. In order to explore the underlying material basis and molecular mechanisms responsible for the anti-breast cancer effects of Ecliptae Herba, we used network pharmacology and experimental verification. UPLC-MS/MS was utilized to identify compounds present in Ecliptae Herba. The active components of Ecliptae Herba and its breast cancer targets were screened using public databases. Hub genes were identified using the STRING and Metascape database. The R software was utilized for visual analysis of GO and KEGG pathways. The affinity of the hub targets for the active ingredients was assessed by molecular docking analysis, which was verified by experimental assessment. A total of 178 targets were obtained from the 10 active components of Ecliptae Herba, while 3431 targets associated with breast cancer were screened. There were 144 intersecting targets between the components and the disease. Targets with a higher degree, namely EGFR and TGFB1, were identified through the hub subnetwork of PPI. GO and KEGG analyses revealed that Ecliptae Herba plays an important role in multiple cancer therapeutic mechanisms. Moreover, molecular docking results showed that the core components had good binding affinity with key targets. Finally, it was confirmed that TGF-β1 might be a potential crucial target of Ecliptae Herba in the treatment of breast cancer by cytological experiments, and the TGF-β1/Smad signaling pathway might be an important pathway for Ecliptae Herba to exert its therapeutic effects. This study elucidated the active ingredients, key targets, and molecular mechanisms of Ecliptae Herba in the treatment of breast cancer, providing a scientific foundation and therapeutic mechanism for the prevention and treatment of breast cancer with Traditional Chinese medicine.

## 1. Introduction

Breast cancer in women has become the most common cancer worldwide. There are an estimated 2.3 million new cases each year, accounting for about 11.7% of all new cancer cases.^[1]^ At present, the primary approach to treating breast cancer involves surgical resection of the lesion in conjunction with radiotherapy and chemotherapy.^[2]^ However, the trauma of surgery and the adverse reactions of radiotherapy and chemotherapy seriously affect the patients’ quality of life.^[3,4]^ Therefore, it is urgent to find more effective drugs with less side effects for breast cancer treatment.

Traditional Chinese medicine (TCM) has been widely used over time. It plays a crucial role in the prophylaxis and cure of breast cancer.^[5]^ Ecliptae Herba is the dry aerial part of Eclipta prostrata L., which belongs to the Asteraceae family.^[6]^ It has a rich historical background in medicinal practices across Asia, South America, and various other nations. It is often used in immune regulation, liver protection, blood lipid reduction, antioxidant properties, and anti-aging effects.^[7–9]^ In recent years, the antitumor effects of Ecliptae Herba have been discovered. Studies have shown that multiple components of Ecliptae Herba have anti-tumor activities.^[10–13]^ Yadav explored the inhibitory effects of Ecliptae Herba ethanol extract on 7 different cancer cell lines and found that the proliferation and migration of breast cancer cells were the most inhibited.^[14]^ However, the mechanism of action of Ecliptae Herba in breast cancer treatment remains unknown. This underscores the significance of conducting additional research and exploration to enhance our understanding and potential application of Ecliptae Herba in the prevention and treatment of breast cancer.

**Figure 1.**
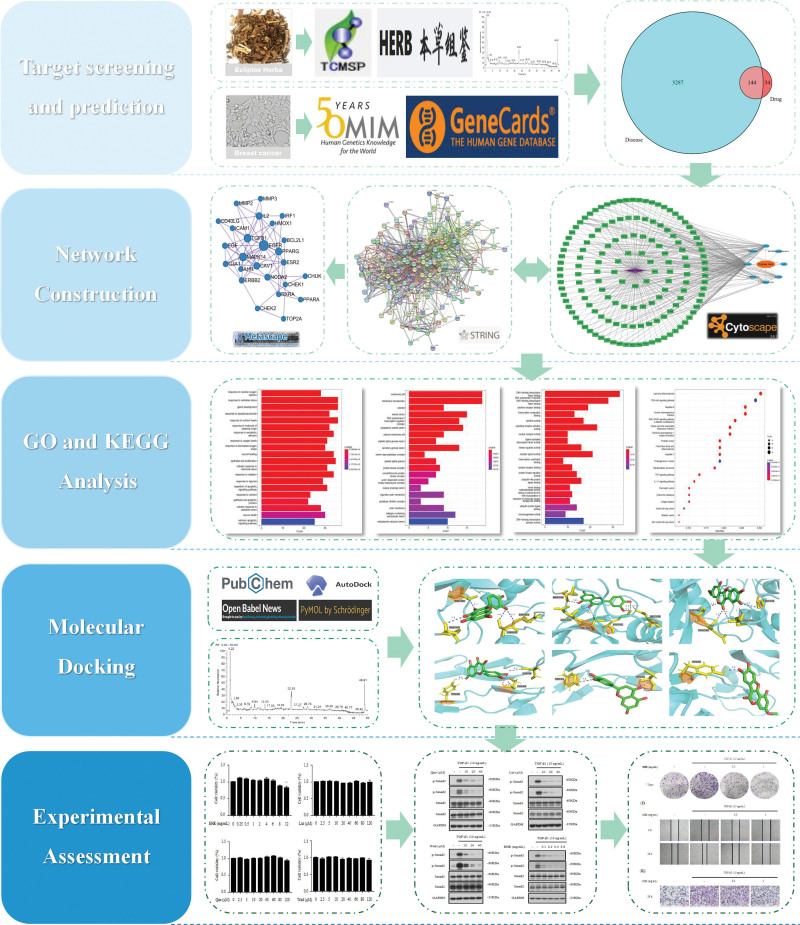
The workflow of network pharmacology analysis and experimental assessment.

Network pharmacology is a discipline that uses network methods to analyze the synergistic relationship of “multi-components, multi-targets, and multi-pathways” between drugs, diseases, and targets,^[15]^ which builds a bridge for the research on the relationship between traditional herbal medicine and modern pharmacology. It is helpful to identify therapeutic targets of the active components to enhance the curative effect and reduce adverse drug reactions.^[16]^ In this study, the effective components and molecular mechanisms of Ecliptae Herba in treating breast cancer were predicted using network pharmacology and molecular docking methods, and the results were verified experimentally, which provided a basis for future research on Ecliptae Herba and its active compounds (Fig. 1).

## 2. Materials and methods

### 2.1. UPLC-MS analysis of the main ingredients in Ecliptae Herba extract

Ecliptae Herba (EH) samples were prepared at a concentration of 5 mg/mL in 50% methanol-water. Centrifugation was performed at 10,000 g for 10 minutes, and the precipitant (methanol: acetonitrile = 1:1) was added to the supernatant to precipitate impurities. Centrifugation was performed again and the supernatant was removed for UPLC-MS analysis. Chromeleon software combined with TCM data processor and relevant literature information were used to identify the components of Ecliptae Herba extract.

UPLC-MS condition: DIONEX Ultimate 3000 UPLC system coupled with Thermo Q EXACTIVE instrument was used to analyze the samples. The mobile phases were (A) water (containing 2mmol/L ammonium formate and 0.1% formic acid) and (B) acetonitrile. The gradient elution condition: 0 to 2 minutes (95%A); 2 to 42 minutes (95%–5%A); 42 to 47 minutes (5%–95%A); 47 to 50 minutes (95%A). Flow rate: 0.25 mL/min. Injection volume: 5 μL.

### 2.2. Screening of Ecliptae Herba components and targets

The chemical compositions of Ecliptae Herba was screened based on the Traditional Chinese Medicine Systems Pharmacology Database and Analysis Platform database (TCMSP, https://tcmsp-e.com/tcmsp.php)^[17]^ and HERB database (http://herb.ac.cn/).^[18]^ In addition, chemical components identified by UPLC-MS were combined. Components with oral bioavailability ≥ 30% and drug-likeness ≥ 0.18 were selected as the potential active components of Ecliptae Herba and their protein targets were searched in the TCMSP database.^[19]^ Then, the selected targets were converted into UniProt ID format using UniProt database (http://www.uniprot.org/).^[20]^

### 2.3. Candidate targets collection of breast cancer

Based on the GeneCards (https://www.genecards.org/)^[21]^ and OMIM (https://omim.org/) databases,^[22]^ “Breast cancer” was used as the search term to obtain relevant targets. The retrieval results from the 2 databases were merged to remove duplicates and obtain information on breast cancer-related targets. A Venn diagram of the intersection targets of Ecliptae Herba and breast cancer was plotted using R software (https://www.r-project.org/).^[23]^

### 2.4. Network construction and hub genes screening

STRING (https://cn.string-db.org/)^[24]^ was used to obtain the protein-protein interaction (PPI) network of the intersecting genes. Set the species source to “Homo sapiens” and set the “minimum required interaction score” to 0.700. Then, the PPI network was divided to screen out central subnetwork and hub genes related to breast cancer using Metascape (https://metascape.org/).^[25]^

Cytoscape3.9.1 software (http://www.cytoscape.org/)^[26]^ was used to map the regulatory network of “Drug-Component-Target-Disease.” The nodes represent drugs, components, targets, and diseases in this network. Lines represent interactions between nodes. Degree is an important parameter that represents the number of nodes that directly interact with a node.^[27]^ The degree value between network nodes was calculated, and the active ingredients with higher degree were selected as target components for the subsequent experiments.

### 2.5. GO enrichment analysis and KEGG pathway analysis

Gene ontology (GO) enrichment analysis and Kyoto encyclopedia of genes and genomes (KEGG) pathway enrichment analysis of the anti-breast cancer targets of Ecliptae Herba were performed by R software to clarify the role of target proteins in gene function and signaling pathways.^[28]^ GO enrichment analysis includes biological processes (BP), cellular components (CC), and molecular functions. The GO term and KEGG pathway were considered statistically significant at *P* < .05. The top 20 enriched entries for each item are plotted as bar or bubble plots. Cytoscape was used to construct a network diagram to visualize target-pathway relationships.

### 2.6. Molecular docking

The 3D structure of molecular ligands were downloaded from the PubChem database (https://pubchem.ncbi.nlm.nih.gov/)^[29]^ and converted to pdb format using OpenBabel3.1.1 (https://openbabel.org/). The crystal structure of the hub target proteins were downloaded from the RCSB Protein Data Bank database (https://www.rcsb.org/)^[30]^ and imported into PyMOL2.4.0 software to remove the original ligands and HOH. Next, hydrogenation and charge calculations were performed on target proteins using AutoDock Tools 1.5.7, and exported in pdbqt format. Finally, molecular docking was performed using AutoDock Vina and the results were visualized with PyMOL.

The docking affinity was used to evaluate the ability of receptor to ligand. A binding affinity of <0 kcal/mol indicates that the receptor and ligand are able to bind spontaneously.^[31]^ The lower the docking affinity, the higher binding possibility and the more stable the binding conformation. When the docking affinity is less than −5 kcal/mol, it demonstrates good binding potential between the receptor and ligand.^[32]^

### 2.7. Experimental assessment

#### 2.7.1. Reagents and antibodies.

Reagents: Quercetin (Que, MedChemExpress, HY-18085), Luteolin (Lut, MedChemExpress, HY-N0162), Wedelolactone (Wed, MedChemExpress, HY-N0551), Human TGF-β1 (PeproTech, AF-100-21C-10), Human EGF (PeproTech, AF-100-15), Cell Counting Kit-8 (CCK-8, Dojindo, CK04), DMEM (Macgene, CM10013), 0.05% Trypsin-EDTA (Macgene, CC017.2), Fetal Bovine Serum (FBS, VivaCell, C04001-500, Shanghai, China), Crystal Violet (TargetMol, T1343L, USA), VitroGel Hydrogel Matrix (THE WELL, VHM01).

Antibodies: p-Smad3 (abcam, ab52903), p-Smad2 (abcam, ab280888), Smad3 (abcam, ab40854), Smad2 (abcam, ab40855), p-EGFR (abcam, ab40815), EGFR (abcam, ab52894), GAPDH (Proteintech, 60004-1-Ig).

#### 2.7.2. Cell culture.

MDA-MB-231 (hereinafter referred to as MB231) were provided by Dr Tao Li of the National Center of Biomedical Analysis (Beijing, China). MB231 cells were cultured in DMEM with 10% FBS and 1% penicillin-streptomycin in a cell incubator at 37°C and 5%CO_2_, and experiments were performed when cells were in a good growth state.

#### 2.7.3. Cell viability assay.

MB231 at 1.5 × 10^5^ cells/mL were seeded in 96-well cell culture plate. After cells attached to the wall, they were treated with Ecliptae Herba (0, 0.25, 0.5, 1, 2, 4, 6, 8, and 12 mg/mL) for 24 hours or treated with Que (0, 2.5, 5, 10, 20, 40, 60, 80, and 120 μM), Lut (0, 2.5, 5, 10, 20, 40, 60, 80, and 120 μM), Wed (0, 2.5, 5, 10, 20, 40, 60, 80, and 120 μM) for 6 hours. Then, CCK-8 was added in the dark condition. Then cells were further cultured in the incubator for 1 hour. The OD value at 450 nm of each group was measured by microplate reader.

#### 2.7.4. Cell colony formation assay.

MB231 were cultured in cell culture plates at a concentration of 1 × 10^3^ cells/mL and gently shaken to evenly disperse the culture plates. The next day, cells were added Ecliptae Herba (0.5 and 1 mg/mL) and TGF-β1 (10 ng/mL, the concentration of TGF-β1 used in all the following experiments was 10 ng/mL) for 7 days at 37°C. Then, MB231 were fixed in a cell fixative solution and stained with crystal violet for 15 minutes and the results were photographed.

#### 2.7.5. Cell wound-healing assay.

Cell Culture-Inserts were fixed in the cell culture plates. MB231 in a good growth condition were seeded in Culture-Inserts. After cells attachment, Culture-Inserts were removed and cells were treated with Ecliptae Herba (0.5 and 1 mg/mL) and TGF-β1 for 24 hours at 37°C. MB231 were photographed under an inverted microscope at 0 and 24 hours.

#### 2.7.6. Transwell invasion assay.

The VitroGel Hydrogel Matrix was diluted with DMEM and evenly spread to upper chamber polycarbonate membranes of Transwell plates with 100 μL per well. After matrix gelation, cell suspension and Ecliptae Herba (0.5 and 1 mg/mL) were added to the upper chamber. DMEM with or without TGF-β1 was added to the lower chamber. After 24 hours, the invasive cells were fixed in cell fixative solution and stained with crystal violet for 15 minutes. The cells were observed under a microscope.

#### 2.7.7. Western Blot assay.

MB231 in a good growth condition were treated with EH, Que, Lut or Wed for 1 hour, followed by TGF-β1 for 3 hours. MB231 were pretreated with EH and the 3 ingredients separately, followed by EGF (10 ng/mL) for 30 minutes. According to the method described in literature,^[33]^ cell lysates were collected for Western Blot assay. The samples were separated by electrophoresis on a 10% sodium dodecyl sulfate-polyacrylamide gel, transferred to a polyvinylidene difluoride membrane, and sealed with 5% skim milk powder for 1 hour. Then, we added corresponding primary antibodies including p-Smad3, p-Smad2, Smad3, Smad2, p-EGFR, EGFR, and GAPDH, respectively. This was followed by incubation with secondary antibodies. GAPDH was used as a control.

#### 2.7.8. Statistical analysis.

Graphpad prism 7.00 was used for statistical analysis. The data were expressed by mean ± standard error of mean (mean ± SEM). One-way analysis of variance (ANOVA) was used to compare multiple groups of data. *P* < .05 was considered statistically significant.

## 3. Results

### 3.1. UPLC-MS/MS analysis of the main components in Ecliptae Herba

**Figure 2.**
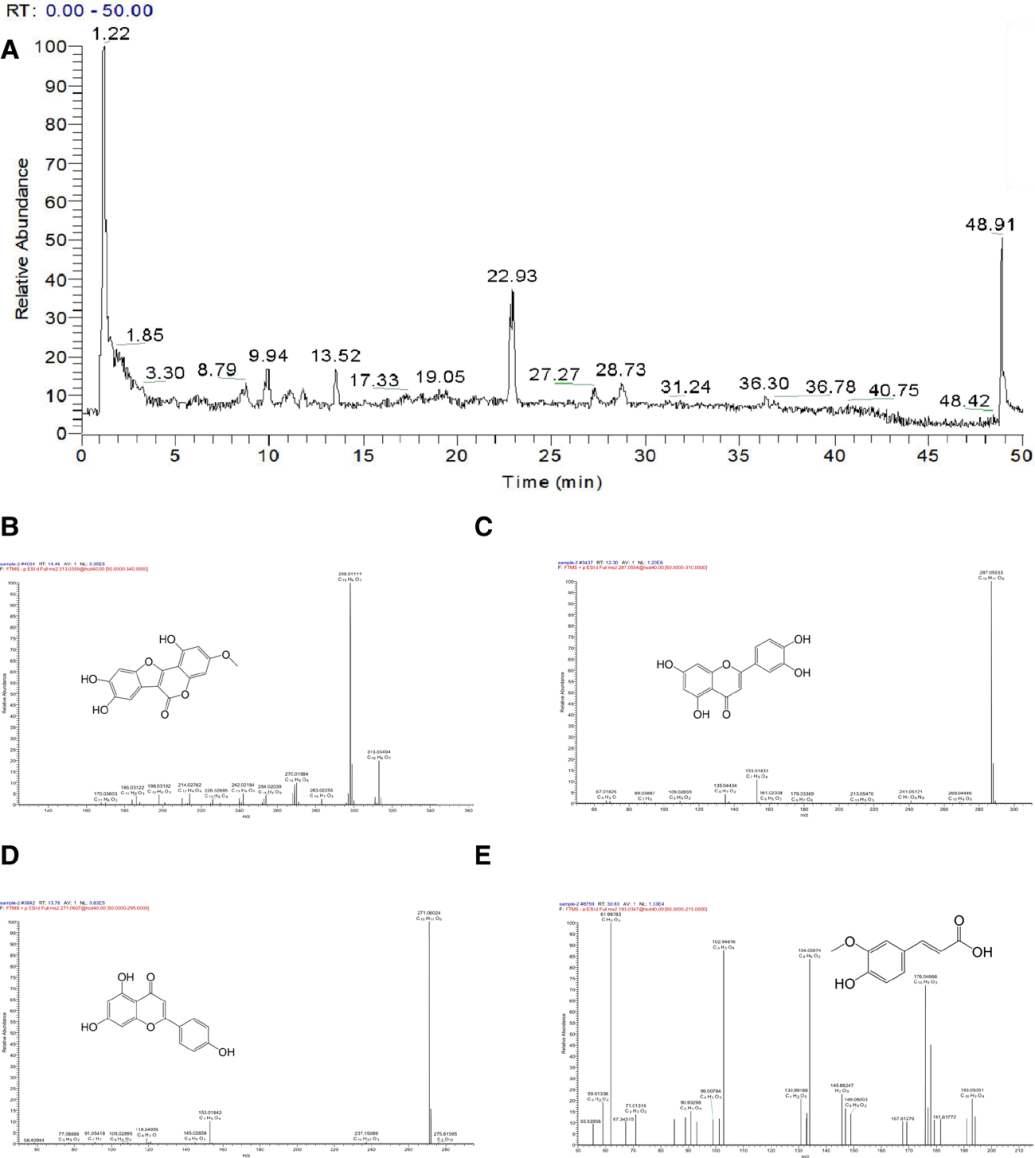
The chemical compositions of Ecliptae Herba (EH) were identified by UPLC-MS. (A) Total ion flow chromatogram of EH in positive ion mode (NL: 5.85E8). Component secondary mass spectrograms of Ecliptae Herba including (B) Wedelolactone, (C) Luteolin, (D) Apigenin, and (E) Ferulic acid.

High-resolution information on the Ecliptae Herba samples was acquired through UPLC-MS/MS using a TCM data processor. Combined with the literatures,^[34,35]^ the chemical constituents of Ecliptae Herba were identified using a multi-parameter database based on neutral mass number, chromatographic retention time and MS/MS fragmentation data. The total ion flow chromatogram and secondary mass spectrums of some components are shown in Figure 2 and the results are shown in Table 1.

**Table 1:** Identification of chemical constituents in Ecliptae Herba.

No.	Predictive compounds	Molecular formula	*m/z* (expected)	*m/z* [delta (ppm)]	t_R_/min
1	3,5-Dihydroxybenzoic acid	C7H6O4	155.03	4.23	3.94
2	1-Caffeoylquinic acid	C16H18O9	353.09	−4.70	6.55
3	Caffeic acid	C9H8O4	179.03	−4.90	7.05
4	Cyanidin 3-O-glucoside	C21H21O11	450.12	−3.86	8.79
5	Astragalin	C21H20O11	449.11	2.74	10.47
6	Demethylwedelolactone	C15H8O7	299.02	−6.58	10.59
7	Quercetin	C15H10O7	303.05	2.81	10.87
8	Diosmetin-7-O-beta-D-glucopyranoside	C22H22O11	463.12	2.27	10.97
9	Apigenin-7-glucuronide	C21H18O11	447.09	2.26	11.67
10	1,4-Dicaffeoylquinic acid	C25H24O12	517.13	2.56	11.95
11	Luteolin	C15H10O6	287.06	3.24	12.39
12	Myricetin	C15H10O8	317.03	−3.13	12.82
13	Abscisic acid	C15H20O4	265.14	2.27	13.52
14	Apigenin	C15H10O5	271.06	2.32	13.62
15	Linarin	C28H32O14	593.19	2.34	14.00
16	Wedelolactone	C16H10O7	313.04	−2.66	14.39
17	Ferulic acid	C10H10O4	195.07	3.97	14.52
18	Butin	C15H12O5	273.08	2.10	14.99
19	Ecliptasaponin A	C36H58O9	633.40	−5.48	20.60
20	L-Asparagine	C4H8N2O3	133.06	−1.79	22.93
21	Ursolic acid	C30H48O3	457.37	−2.95	33.75
22	Fraxetin	C10H8O5	209.04	−5.84	48.94

### 3.2. Acquisition of Ecliptae Herba active ingredients and the main protein targets

A total of 78 components of Ecliptae Herba were obtained by searching the HERB and TCMSP databases, and 22 components were identified by UPLC-MS analysis. According to the criteria of oral bioavailability ≥ 30% and drug-likeness ≥ 0.18, 10 potential active ingredients and 178 target proteins were obtained. Basic information of the active ingredients is presented in Table 2.

**Table 2:** Basic information of Ecliptae Herba compositions.

Mol ID	Molecule name	OB (≥30%)	DL(≥0.18)
MOL001790	Linarin	39.84	0.71
MOL001689	Acacetin	34.97	0.24
MOL002975	Butin	69.94	0.21
MOL003378	1,3,8,9-tetrahydroxybenzofurano[3,2-c] chromen-6-one	33.94	0.43
MOL003389	3’-O-Methylorobol	57.41	0.27
MOL003398	Pratensein	39.06	0.28
MOL003402	Demethylwedelolactone	72.13	0.43
MOL003404	Wedelolactone	49.6	0.48
MOL000006	Luteolin	36.16	0.25
MOL000098	Quercetin	46.43	0.28

DL = drug likeness, OB = oral bioavailability.

### 3.3. Acquisition of breast cancer target genes

In the GeneCards and OMIM databases, 16,032 and 217 breast cancer-related genes were screened respectively. The duplicated genes in the 2 databases were merged and 3431 genes were selected for further analysis.

### 3.4. PPI network construction and hub genes screening

**Figure 3.**
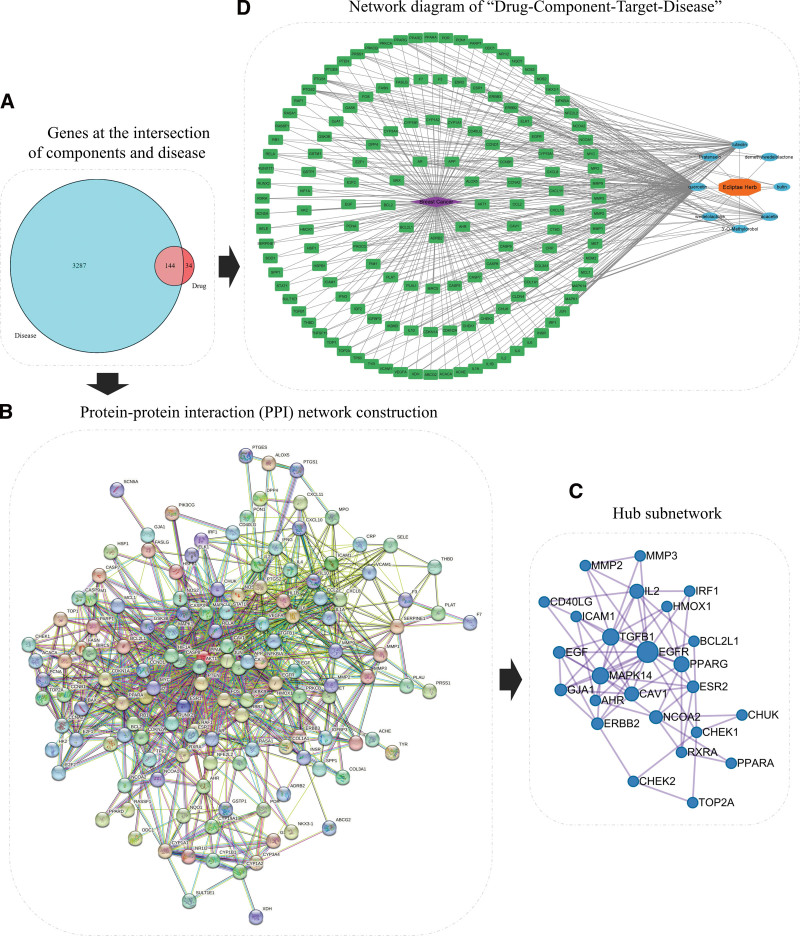
Protein-protein interaction (PPI) network and “Drug-Component-Target-Disease” network construction. (A) Venn diagram of intersection targets of Ecliptae Herba components and breast cancer drawn by R software. (B) The PPI network obtained by STRING database. (C) Hub genes of PPI network analyzed by Metascape database. (D) The network of “Drug-Component-Target-Disease” obtained by Cytoscape software. PPI = protein-protein interaction.

The common targets of components and disease were taken to obtain 144 intersecting targets. This was visualized using R software (Fig. 3A). Intersection targets were imported into the STRING database to obtain a PPI network with 144 nodes and 1027 edges (Fig. 3B). Then, the PPI network was divided into modules using the Metascape. The hub genes highly associated with tumors were EGFR, TGFB1, MAPK14, PPARG and IL2 with high degree (Fig. 3C). EGFR (Degree:14) and TGFB1 (Degree:10) with the highest degrees were selected for subsequent molecular docking analysis and experimental exploration.

### 3.5. Network diagram of “Drug-Component-Target-Disease”

Cytoscape software was applied to draw the “Drug-Component-Target-Disease” regulatory network of Ecliptae Herba for breast cancer treatment. The network contains 154 nodes and 386 edges (Fig. 3D). The components of degree > 5 are Quercetin (Que), Luteolin (Lut), Acacetin, Pratensein, 3’-O-methylorobol, and Wedelolactone (Wed), indicating that these components can interact with more disease targets and may be important components of Ecliptae Herba in the prevention and treatment of breast cancer. However, 3 components (Acacetin, Pratensein, and 3’-O-methylorobol) were not detected in the UPLC-MS analysis. Combined with the literatures,^[14,34,35]^ we speculated that their content might be very low in Ecliptae Herba. Therefore, we did not consider these to be the main components of Ecliptae Herba that exert its medicinal effects. Que, Lut, and Wed were selected for the molecular docking and experimental verification.

### 3.6. GO enrichment and KEGG pathway analyses

**Figure 4.**
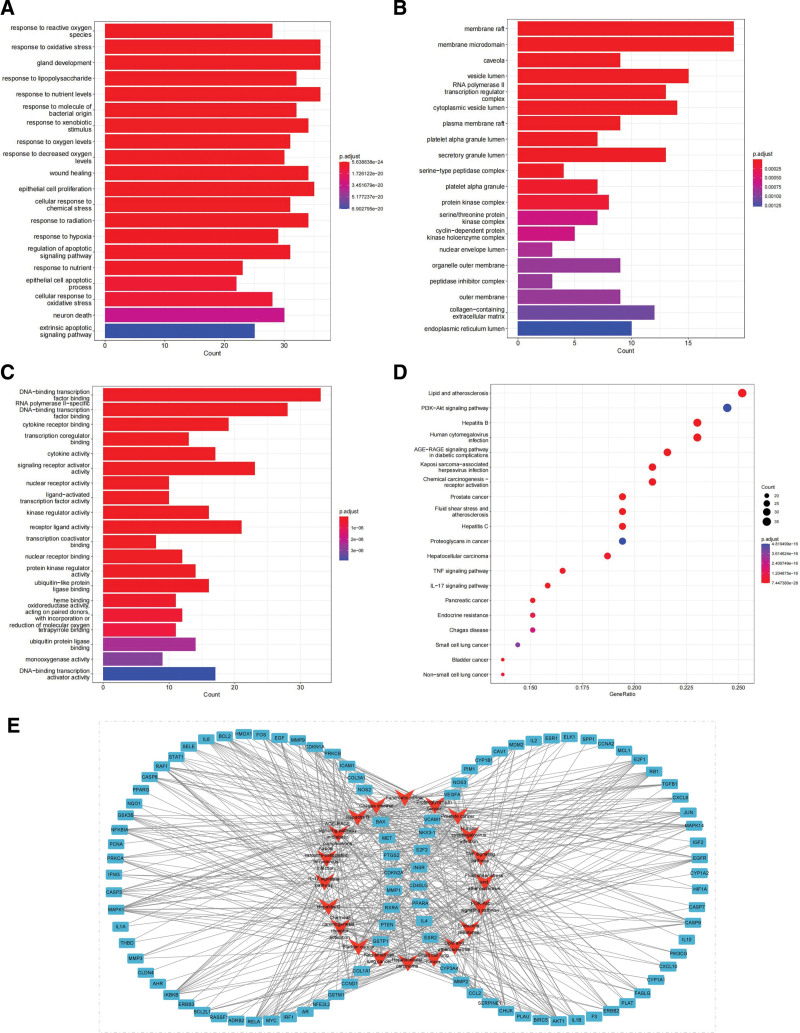
GO enrichment and KEGG pathway analyses. Bar plots show (A) biological processes, (B) cell components, and (C) molecular functions of GO enrichment analysis. (D) Bubble plot shows KEGG pathway enrichment analysis. (E) Target-pathway network diagram of Ecliptae Herba in the treatment of breast cancer. GO = Gene ontology, KEGG = Kyoto encyclopedia of genes and genomes.

R software was used to perform GO enrichment analysis of target genes in PPI network. There are 2152 BP, mainly involved in response to reactive oxygen species, oxidative stress, gland development, lipopolysaccharide, and nutrient levels (Fig. 4A). The cell components have 51 items, including the membrane raft, membrane microdomain, caveola, vesicle lumen, and RNA polymerase II transcription regulator complex (Fig. 4B). A total of 159 items are involved in molecular functions, including DNA-binding transcription factor binding, RNA polymerase II-specific DNA-binding transcription factor binding and cytokine receptor binding (Fig. 4C). KEGG analysis identified 159 signaling pathways (*P* < .05). It mainly included the AGE-RAGE signaling pathway in diabetic complications, prostate cancer, Hepatitis B, lipid and atherosclerosis and bladder cancer, which were significantly enriched (Fig. 4D). The gene target-pathway signal network plotted by Cytoscape software is shown in Figure 4E.

### 3.7. Molecular docking

**Figure 5.**
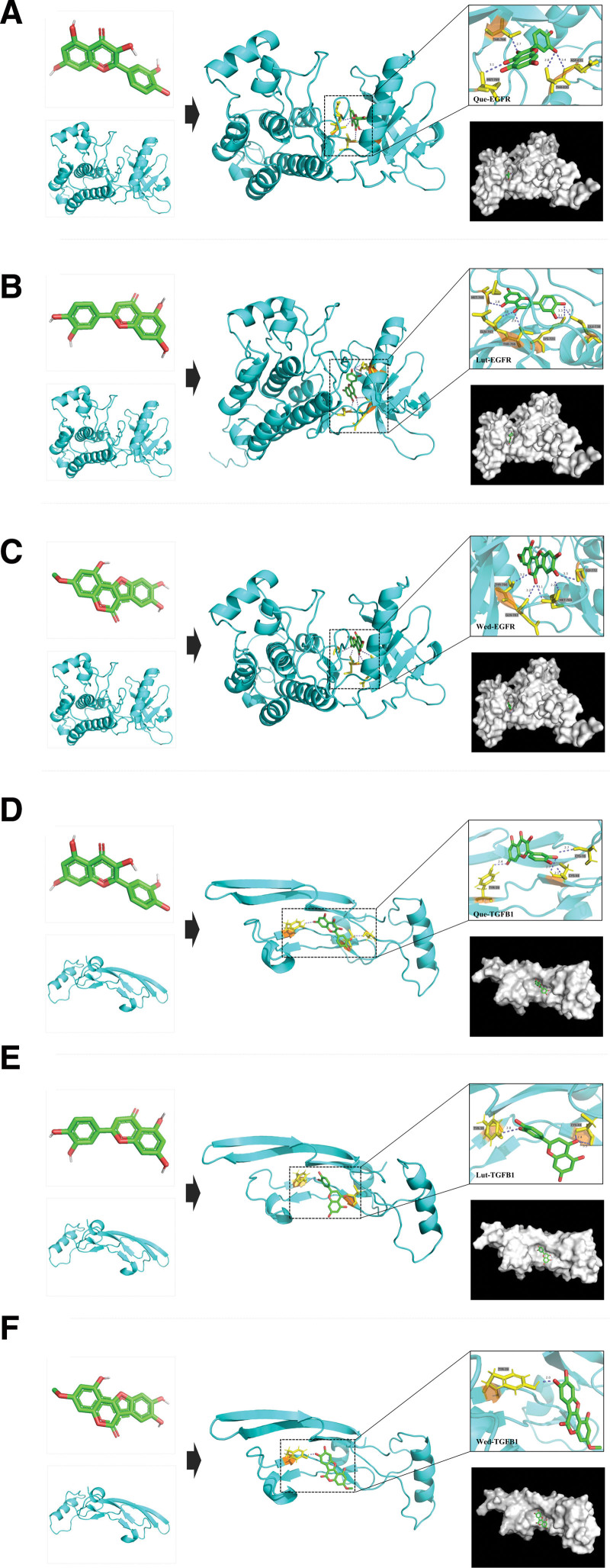
Molecular docking of the main compounds and target proteins. Compounds (green), target proteins (cyan), amino acid residues (yellow), hydrogen bonds (slate). (A) Quercetin (Que), (B) Luteolin (Lut), and (C) Wedelolactone (Wed) are shown interacting with the target protein of EGFR. (D) Que, (E) Lut, and (F) Wed are shown interacting with the target protein of TGFB1.

Que, Lut and Wed were selected for the molecular docking analysis of the hub genes EGFR and TGFB1 by AutoDock Vina. The results indicated that Que, Lut, and Wed, the active components of Ecliptae Herba, showed good binding affinity to EGFR and TGFB1 (affinity < −5 kcal/mol, Table 3). Among them, Wed-EGFR and Que-TGFB1 were the most highly rated. The docking results were analyzed by 3D visualization using PyMOL (Fig. 5A–F).

**Table 3:** Affinity value of the main ligands with receptors.

Target proteins	Compounds	Binding Affinity (kcal/mol)	Interacting residues
EGFR	Quercetin	−7.7	ASP-831, MET-769, THR-766, THR-830
	Luteolin	−7.6	GLN-767, GLU-738, LYS-721, MET-769, THR-766
	Wedelolactone	−7.8	GLN-767, GLY-772, MET-769, THR-766
TGFB1	Quercetin	−6.9	CYS-44, CYS-78, TYR-39
	Luteolin	−6.8	CYS-44, TYR-39
	Wedelolactone	−6.7	TYR-39

### 3.8. Experimental assessment

#### 3.8.1. Cell viability of Ecliptae Herba and its active components in MB231.

**Figure 6.**
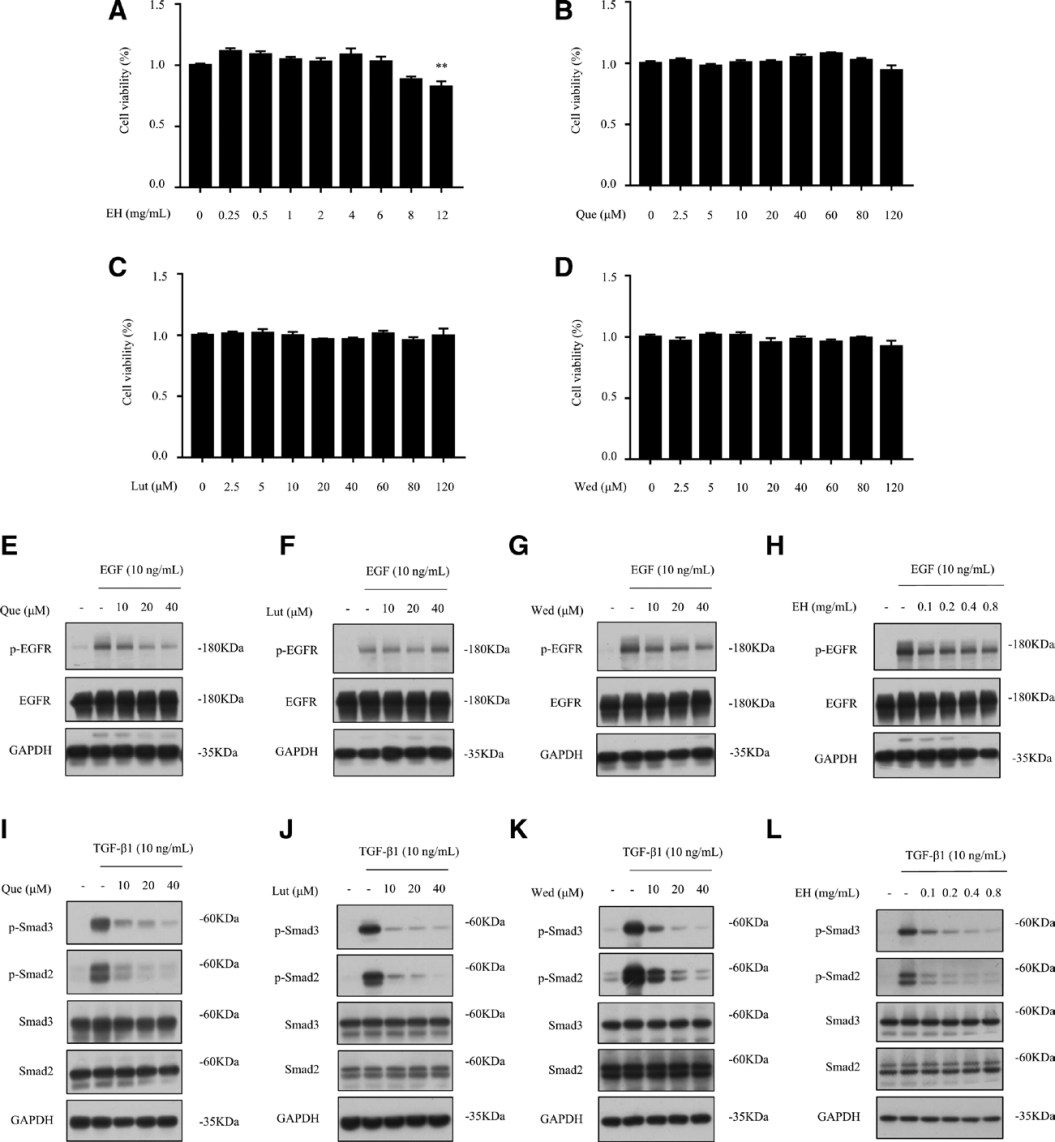
Experimental assessment of Ecliptae Herba and its active ingredients. (A) MB231 was treated with EH (0, 0.25, 0.5, 1, 2, 4, 6, 8, and 12 mg/mL) for 24 h. The cell viability was determined by CCK-8 assay. (B-D) MB231 was treated with Que (0, 2.5, 5, 10, 20, 40, 60, 80, and 120 μM), Lut (0, 2.5, 5, 10, 20, 40, 60, 80, and 120 μM) or Wed (0, 2.5, 5, 10, 20, 40, 60, 80, and 120 μM) for 6 h. The cell viability was determined by CCK-8 assay. (E-H) MB231 was pretreated with EH (0.1, 0.2, 0.4, and 0.8 mg/mL), Que (10, 20, and 40 μM), Lut (10, 20, and 40 μM) or Wed (10, 20, and 40 μM) for 1 h followed by EGF (10 ng/mL) stimulation for 30 min. The expressions of p-EGFR, EGFR and GAPDH were detected by Western Blot assay. (I-L) MB231 was pretreated with EH (0.1, 0.2, 0.4, and 0.8 mg/mL), Que (10, 20, and 40 μM), Lut (10, 20, and 40 μM) or Wed (10, 20, and 40 μM) for 1 h followed by TGF-β1 (10 ng/mL) stimulation for 3 h. The expressions of p-Smad3, p-Smad2, Smad3, Smad2, and GAPDH were detected by Western Blot assay. All data are expressed by means ± SEM. One-way analysis of variance (ANOVA) was used to assess the differences of multiple groups. ^**^P < .01 versus control group.

We examined the cell viability of Ecliptae Herba, Que, Lut and Wed in MB231. The results showed that Ecliptae Herba at the concentration of 0 to 8 mg/mL did not affect the cell viability of MB231 within 24 hours, but had obvious cytotoxicity when the concentration of Ecliptae Herba reached 12 mg/mL (*P* < .01, Fig. 6A). Que, Lut, and Wed at 0 to 120 μM had no significant effect on the cell viability of MB231 within 6 hours (Fig. 6B–D). We chose their safe concentrations for subsequent experiments.

#### 3.8.2. Effects of Ecliptae Herba and its active ingredients on EGFR and TGFB1 related pathways.

Through molecular docking, we found that Que, Lut and Wed have good affinities for EGFR and TGFB1 (TGF-β1), respectively. To verify the effect of Ecliptae Herba and its 3 active ingredients on the core targets, we detected the expression of the relevant proteins by Western Blot assay.

EGFR is a glycoprotein belonging to the tyrosine kinase-type receptor family that is activated by dimerization and autophosphorylation after binding to the EGF ligand. This leads to a phosphorylation cascade that activates downstream pathways and regulates the occurrence and progression of tumors.^[36]^ Therefore, the inhibitory effects of EH, Que, Lut and Wed on the expression of EGF-induced p-EGFR were tested to preliminarily evaluate the role of the active components in regulating breast cancer through EGFR-related signaling pathways. The results showed no significant inhibitory effect on p-EGFR expression (Fig. 6E–H).

Most tumor cells can produce TGF-β1 through autocrine or paracrine signals, resulting in higher TGF-β1 concentrations in tumor tissues than physiological levels. At this time, TGF-β1 often promotes epithelial-mesenchymal transitions (EMT) in tumor cells, leading to tumor invasion and metastasis.^[37]^ In most breast cancers and their metastases, the transformation of TGF-β1 from a tumor suppressor to a tumor promoter is positively correlated with phosphorylated Smad2/3. TGF-β1 induces the transformation of TGF-β by binding to its receptor and modulating the classical Smad pathway. The Smad pathway is an important pathway through which TGF-β1 functions. Hence, we examined the effects of Ecliptae Herba, Que, Lut, and Wed on TGF-β1 induced p-Smad2/3 expression to appraise the function of Ecliptae Herba about regulating breast cancer through the TGF-β1 related signaling pathways. The results proved that all 3 active ingredients could significantly reduce TGF-β1-induced phosphorylation of Smad2 and Smad3 in a concentration-dependent manner (Fig. 6I–K). And then, further experiments showed that Ecliptae Herba can also inhibit the TGF-β1-induced expression of p-Smad2 and p-Smad3 (Fig. 6L).

#### 3.8.3. Ecliptae Herba inhibits proliferation, migration and invasion of MB231 induced by TGF-β1.

Metastasis is an important factor in breast cancer mortality.^[38]^ TGF-β signaling pathway is particularly associated with cancer cell metastasis. Abnormal expression of TGF-β1 can promote the migration and invasion of breast cancer cells.^[39]^ We, therefore, explored the function of Ecliptae Herba on migration and invasion of MB231 induced by TGF-β1.

**Figure 7.**
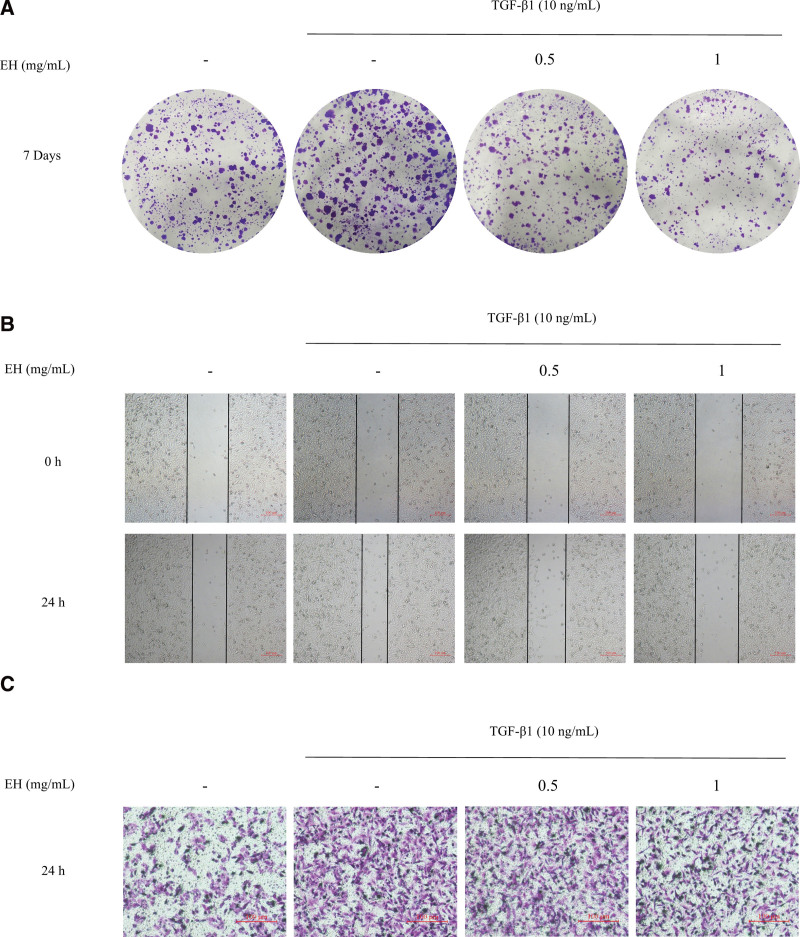
The effect of Ecliptae Herba on TGF-β1 mediated proliferation, migration and invasion of MB231. (A) The EH effect on the proliferation ability of MB231 was detected by cell colony formation assay. (B) The effect of EH on MB231 migration ability was detected by cell wound-healing assay. (C) The effect of EH on MB231 invasion ability was detected by Transwell invasion assay.

First, we used cell colony formation assay to observe the effect of Ecliptae Herba on TGF-β1 induced cell proliferation. The results showed that TGF-β1 significantly enhanced the colony-forming ability of MB231, and Ecliptae Herba could reduce TGF-β1-induced MB231 proliferation (Fig. 7A). After that, the effect of Ecliptae Herba on cell migration was verified by cell wound-healing assay. The experimental results indicated that Ecliptae Herba inhibited the migration of MB231 significantly induced by TGF-β1 (Fig. 7B). We also explored the effect of TGF-β1 and Ecliptae Herba on the invasion of MB231 by Transwell invasion assay. As can be seen from the results, Ecliptae Herba inhibited TGF-β1-induced invasion of MB231 (Fig. 7C).

## 4. Discussion

Breast cancer is the most common malignant tumor in women worldwide.^[40]^ Over the years, with the in-depth research of TCM in breast cancer, Traditional Chinese medicine has gradually shown its advantages and characteristics in reducing the adverse reactions of radiotherapy and chemotherapy, and preventing tumor recurrence and metastasis.^[41]^ It is an effective adjuvant therapy for various cancers.^[42]^ Ecliptae Herba, as a widely used TCM, has been shown to have anti-cancer effects.^[14,43]^ However, the active ingredients, therapeutic targets, and mechanisms of breast cancer treatment have not been elucidated. Network pharmacology can discover key bioactive compounds and effective targets from large amounts of data to systematically and comprehensively evaluate drugs and diseases.^[20]^ In this study, we explored the active components and mechanisms of Ecliptae Herba in the prevention and treatment of breast cancer using network pharmacology, molecular docking and experimental validation.

First, UPLC-MS/MS technology was used to identify and screen 10 active ingredients of Ecliptae Herba against breast cancer combined with the network of “Drug-Component-Target-Disease.” Among the 10 active compounds, the degree of Que, Lut and Wed was high, which could be identified in Ecliptae Herba. Previous studies have shown that Quercetin regulates apoptosis in breast cancer cells by regulating Bcl2/BAX inhibition of the PI3K/Akt pathway.^[44]^ Luteolin inhibits VEGF-induced angiogenesis, thereby preventing progestin-driven breast cancer growth and progression.^[45]^ Wedelolactone suppresses the activity of breast cancer cells by acting as inhibitors of multiple proteases.^[46]^ The studies further suggest that the components might be the main active components of Ecliptae Herba in breast cancer treatment.

From 144 intersection targets of Ecliptae Herba and breast cancer, we then obtained the hub targets EGFR and TGFB1 of Ecliptae Herba for breast cancer treatment by constructing PPI network and MCODE function. The studies found that EGFR is widely expressed in breast cancer.^[47]^ The overexpression of EGFR may promote the progression of triple-negative breast cancer.^[48]^ TGF-β is a secretory cytokine that is highly expressed in tumor microenvironment.^[49,50]^ The overexpression of TGF-β1 is closely related to cell carcinogenesis, tumor neovascularization, epithelial-mesenchymal transition, and distant metastasis.^[51]^ The TGF-β1 signaling pathway is currently recognized as the main signaling pathway to promote tumor metastasis.^[52]^ Consistent with literatures, PPI results confirm that EGFR and TGFB1 may be important targets of Ecliptae Herba for the treatment of breast cancer. We explore the enrichment of the targets of Ecliptae Herba for breast cancer treatment in gene function and signal pathway by GO and KEGG analysis. These targets are involved in multiple signaling pathways, including a variety of cancer pathways, the PI3K-Akt signaling pathway and the TNF signaling pathway, and participate in BP, mainly consist of apoptosis, oxidative reaction and, transcriptional regulation. The results suggest that Ecliptae Herba may have a wide range of biological effects. The identification of these key targets and pathways provides a new basis for the prophylaxis and treatment of breast cancer by Ecliptae Herba.

Ultimately, we selected active components and hub genes for molecular docking. The results verified the strong binding affinity between them. On this basis, the active components and key targets selected by network pharmacology and molecular docking were experimentally validated using MB231. The results of in vitro experiments showed that Ecliptae Herba and its 3 active components significantly inhibited the expression of p-Smad2/3 protein induced by TGF-β1, as well as the proliferation, migration and invasion of MB231, suggesting that Ecliptae Herba has a regulatory effect on the growth and metastasis of breast cancer cells. TGF-β1 may be the key target of Ecliptae Herba in the prevention and treatment of breast cancer, and the classical TGF-β1/Smad signaling pathway may be a vital pathway for Ecliptae Herba to exert its therapeutic effects.

To sum up, this study provides preliminary insight into the therapeutic efficacy and underlying molecular mechanisms of Ecliptae Herba in the treatment of breast cancer based on network pharmacology and experimental validation. Based on our results, a variety of ingredients in Ecliptae Herba may act synergistically to treat breast cancer through multiple targets and signaling pathways. However, this study was only verified by in vitro experiments based on network pharmacology analysis, and further clinical trials are needed to verify the therapeutic effect of Ecliptae Herba on breast cancer.

## Author contributions

**Conceptualization:** Hui Li, Junling Cao, Zhaofang Bai.

**Funding acquisition:** Shuanglin Qin, Zhaofang Bai.

**Methodology:** Hui Li, Wei Shi, Tingming Shen, Siwen Hui, Manting Hou, Ziying Wei.

**Project administration:** Zhaofang Bai, Junling Cao.

**Software:** Wei Shi.

**Writing – original draft:** Hui Li.

**Writing – review & editing:** Ziying Wei, Shuanglin Qin.
